# The effect of maternity waiting homes on perinatal mortality is inconclusive: a critical appraisal of existing evidence from Sub-Saharan Africa

**DOI:** 10.1186/s13104-021-05501-2

**Published:** 2021-03-09

**Authors:** Jaameeta Kurji, Kristy Hackett, Kayli Wild, Zohra Lassi

**Affiliations:** 1 School of Epidemiology & Public Health, University of Ottawa, 600 Peter Morand Crescent, Ottawa, ON K1G 5Z3 Canada; 2 Harvard T.H. Chan School of Public Health, 677 Huntington Ave, Boston, MA 02115 USA; 3 Judith Lumley Centre and Institute for Human Security & Social Change, La Trobe University, Plenty Road, Bundoora, Melbourne, 3086 Australia; 4 Robinson Research Institute, Adelaide Medical School, The University of Adelaide, Helen Mayo North, 30 Frome Street, Adelaide, Australia

**Keywords:** Maternity waiting homes, Perinatal mortality, Stillbirths, Meta-analysis, Sub-Saharan Africa, Heterogeneity, Observational studies, Random-effects models

## Abstract

**Objectives:**

To assess the appropriateness of the statistical methodology used in a recent meta-analysis investigating the effect of maternity waiting homes (MWHs) on perinatal mortality in Sub-Saharan Africa.

**Results:**

A recent meta-analysis published in BMC Research Notes used a fixed-effect model to generate an unadjusted summary estimate of the effectiveness of MWHs in reducing perinatal mortality in Africa using ten observational studies (pooled odds ratio 0.15, 95% confidence interval 0.14–0.17). The authors concluded that MWHs reduce perinatal mortality by over 80% and should be incorporated into routine maternal health care services. In the present article, we illustrate that due to the contextual and methodological heterogeneity present in existing studies, the authors’ conclusions about the effectiveness of MWHs in reducing perinatal mortality were likely overstated. Additionally, we argue that because of the selection bias and confounding inherent in observational studies, unadjusted pooled estimates provide little causal evidence for effectiveness. Additional studies with robust designs are required before an appropriately designed meta-analysis can be conducted; until then, the ability to draw causal inferences regarding the effectiveness of MWHs in reducing perinatal mortality is limited.

## Introduction

There is renewed interest in maternity waiting homes (MWHs) as a strategy to increase facility-based obstetric care and reduce maternal and perinatal mortality. MWHs provide temporary accommodation near a health facility prior to birth for women with high-risk pregnancies and/or living far away from health facilities [1]. Several African and Asian countries are investing in MWH scale-up as part of their national health strategies [2–5].

While observational studies have reported some benefits [6–8] there is still insufficient evidence that MWHs reduce mortality [3, 9] or impact newborn outcomes [10]. The quality of available evidence is also low, yet a recently published meta-analysis has drawn strong conclusions in favour of MWHs. An 82.5% reduction in perinatal mortality was attributed to MWH use and consequently, the authors recommend that “all pregnant women be admitted to MWHs before delivery” [11]. This review has been cited repeatedly to advocate MWH use [12–19] despite limitations in the original studies and the review. In this research note, we critically assess the analytic approach employed in this meta-analysis [11] and discuss important considerations when pooling observational data on complex interventions such as MWHs.

## Main text

### Features of the recent meta-analysis on MWHs and perinatal mortality

The meta-analysis by Bekele and colleagues included ten observational studies from six countries [7, 8, 20–27] after 31% (n = 73/236) were excluded because full texts were unavailable [11]. Most of these studies included women who delivered at hospitals offering some level of comprehensive emergency obstetric care [7, 8, 20, 21, 23, 26, 27]. The number of perinatal deaths abstracted for MWH users and women admitted directly to hospitals were reported [11], but there were abstraction errors for two studies [21, 27] and some overlap in data from two studies conducted at Attat Hospital in Ethiopia [8, 24]. Three studies [7, 8, 23] reported stillbirths but not early neonatal deaths and in two others it was difficult to distinguish outcomes for MWH users and non-users [22, 26]. The authors used a fixed-effect model to generate an unadjusted pooled odds ratio estimating the association between MWH use and perinatal mortality. The authors reported conducting sub-group analyses by study design due to the high degree of heterogeneity detected (I^2^ = 97%), but no sub-group estimates were reported or discussed [11].

### Methodological considerations

#### Choice of model for meta-analysis of complex interventions

Decisions about which statistical model to use in a meta-analysis depends on the type of effect expected and the goal of the analysis [28]. Using a fixed-effect model conveys the belief that there is one common true effect size estimated by all individual studies, and that differences in observed effect sizes are a result of sampling error [28–30]. When a fixed-effect model is used, the goal is not to extrapolate findings beyond the included set of studies [28, 31]. In contrast, random-effects models are suitable when a distribution of true effects exists, and included studies represent a random sample of possibilities; in this case, findings may be generalized to other similar scenarios [29].

Heterogeneity is the variability in true effects underlying different studies [32, 33]. The I^2^ statistic (indicates the proportion of variance in observed effects due to variance in true effects and is a “measure of inconsistency”) [32, 33] is often used to decide whether sufficient heterogeneity exists to run a random-effects model but this is not recommended as it has low power [28]. What may be more useful is to assess whether it is likely that studies included are “functionally identical” [29] as assumed under a fixed-effect model. Widespread differences in participant characteristics, intervention designs, settings and outcomes, make the absence of heterogeneity unlikely [28, 33, 34]. Public health interventions are even less likely to be homogenous; they often have interacting components targeting multiple groups, accommodate flexible delivery, and are embedded within complex systems [35]. Given the considerable variation in MWH implementation [36] random-effects models are likely more suitable for meta-analyses involving MWHs.

Alone, however, the estimated mean effect provides an incomplete picture [37] as how effect sizes vary under different conditions and populations is often of interest [38]. With sufficient numbers of studies, sub-group analysis within a few important, pre-specified subgroups (to avoid issues with multiple testing) [28, 39] is one way to explore heterogeneity. Results need to be interpreted cautiously due to the observational nature of the analysis [30].

Finally, in fixed-effect models, larger studies are weighted more heavily [30] as they have smaller sampling error and higher precision. The pooled estimate reported by Bekele et al. [11] was, thus, largely influenced by one study [8] (weight: ~ 74%). In random-effects models, each study provides unique information about the distribution of true effect sizes, therefore weighting is more equivalent [29].

#### Methodology for the present study

In light of the methodological considerations outlined above, we sought to critically assess the methodology employed by Bekele and colleagues, and explore whether heterogeneity may be better accounted for using a random-effects model. For illustrative purposes, we re-abstracted information from the seven studies [7, 8, 20, 21, 23, 25, 27] from the review that had appropriate data available, as well as three additional eligible studies [40–42] identified from reference lists (Table 1). We calculated a summary estimate in Review Manager version 5.4 using a random-effects model for stillbirths and perinatal mortality separately, using unadjusted outcome events reported for MWH users and women directly admitted to hospital.

**Table 1:** Summary of characteristics of the eleven studies included in the present study

Author (year)	Country	Design and data sources	Facility level, location, obstetric care and managing authority	Type of comparison group	MWH admission criteria and (% MWH use)	Community outreach	Health worker monitoring of users	Study quality^d^
Braat et al (2018)	Ethiopia	Hospital-based retrospective cohort. Hospital records and hospital-based survey	Rural NGO hospital providing CEmOC	Direct admissions^a,b^	Risk factors, distance (34%)	Well established program set up by Attat hospital	None at MWH, users expected to attend routine ANC clinic at hospital	Selection bias possibly present. No adjustment for confounders. Sample size adequacy uncertain. Findings limited to women using hospital-level care
Chandramohan et al. (1995)	Zimbabwe	Hospital based cohort. Labour ward logbook	Rural government referral hospital with doctors on staff to handle complicated births	Direct admissions	Focus on risk factors, but any pregnant woman permitted to stay (35%)	No information	Weekly examination by midwife at MWH	Sample size adequacy uncertain. Findings limited to women using hospital-level care
Fogliati et al. (2017)	Tanzania	Cross-sectional hospital study. Survey and patient records	Rural NGO hospital providing CEmOC	Direct admissions	No information (31%)	No information	No information	No adjustment for confounders. Sample size adequacy uncertain. Uncertainty around comparability of risk profile between groups
Kelly et al. (2010)	Ethiopia	Hospital-based retrospective cohort. Delivery records	Rural NGO hospital providing CEmOC	Direct admissions	Risk factors (average: 28%, range 25%-56%)	Hospital program to train TBAs and CHWs to identify and refer pregnant women	None at MWH, users expected to attend routine ANC clinic at hospital	Descriptive comparison of perinatal outcomes. Uncertainty around comparability of groups. No information on data source completeness
Gaym et al. (2012)^b^	Ethiopia	Hospital-based retrospective cohort. No information	Rural NGO hospital, no information on obstetric care capacity	Direct admissions	No information (10%)	No information	No information	Descriptive comparison of perinatal outcomes between users and non-users that was not the primary aim. Selection bias, sample size adequacy and group comparability all uncertain
Meshesha et al. (2017)	Ethiopia	Hospital-based study with unclear design. Delivery records	Rural government general hospital. No information about obstetric care capacity	Direct admissions	No information (17%)	No information	No information	Descriptive results limited to women able to access facility-based obstetric care. Inadequate reporting makes it difficult to assess the extent of misclassification bias and selection bias
Millard et al. (1991)	Zimbabwe	Hospital-based retrospective cohort. Hospital records	Rural NGO hospital with C-section services	Direct admissions	Focus on risk factors and distance, but any pregnant woman permitted to stay (60%)	No information	No information	Older study with inadequate reporting presenting descriptive results of hospital-based sample. Comparability of group risk profile uncertain
Singh et al. (2016)	Malawi	Hospital-based cross-sectional study. Interviews and intake/discharge forms	2 sites—rural government hospital and urban health centre. No information on obstetric care capacity	Direct admissions	Focus on risk factors and distance, but any pregnant woman permitted to stay (N/A)	Safe Motherhood project in study district includes community education to increase use of services including MWHs	No information	Descriptive results limited to women able to access facility-based obstetric care. Inadequate reporting makes it difficult to assess the extent of misclassification bias and selection bias
Tumwine et al. (1996)	Zimbabwe	Hospital-based retrospective cohort. No information	Rural NGO hospital with C-section services	Direct admissions	No information (27%)	No information	Unclear	Older study with inadequate reporting presenting descriptive results of hospital-based sample. Comparability of group risk profile uncertain
van Lonkhuijzen et al. (2003)	Zambia	Hospital-based retrospective cohort. Surveys	Rural NGO hospital with C-section services	Direct admissions	No information	No information	None at MWH, users expected to attend routine ANC clinic at hospital	Descriptive results limited to women able to access facility-based obstetric care. Inadequate reporting makes it difficult to assess the extent of misclassification bias and selection bias

*CHW* community health worker, *C-section* Caesarean section, *NGO* non-governmental organization, *TBA* traditional birth attendant

^a^Direct admission constitute women who delivered at the health facility but did not stay at the MWH prior to delivery

^b^Braat et al. had two types of comparison groups – direct admissions from Attat hospital and outcomes from women at Butajira hospital that did not have an MWH. Data used here is for Attat non-users only

^c^Perinatal outcomes and MWH use in 2010 reported for Saint Luke’s Hospital in Wolisso, South-west Shoa Zone in Oromiya Region

^d^The AXIS tool for quality appraisal of cross-sectional studies [46] was used to guide rapid review of study quality

To explore heterogeneity, we conducted sub-group analysis for stillbirths to demonstrate how country and type of managing authority may change effect estimates. While no definitive conclusions can be made, the results provide insight into sources of heterogeneity.

### Random effects model findings and implications

**Fig. 1:**
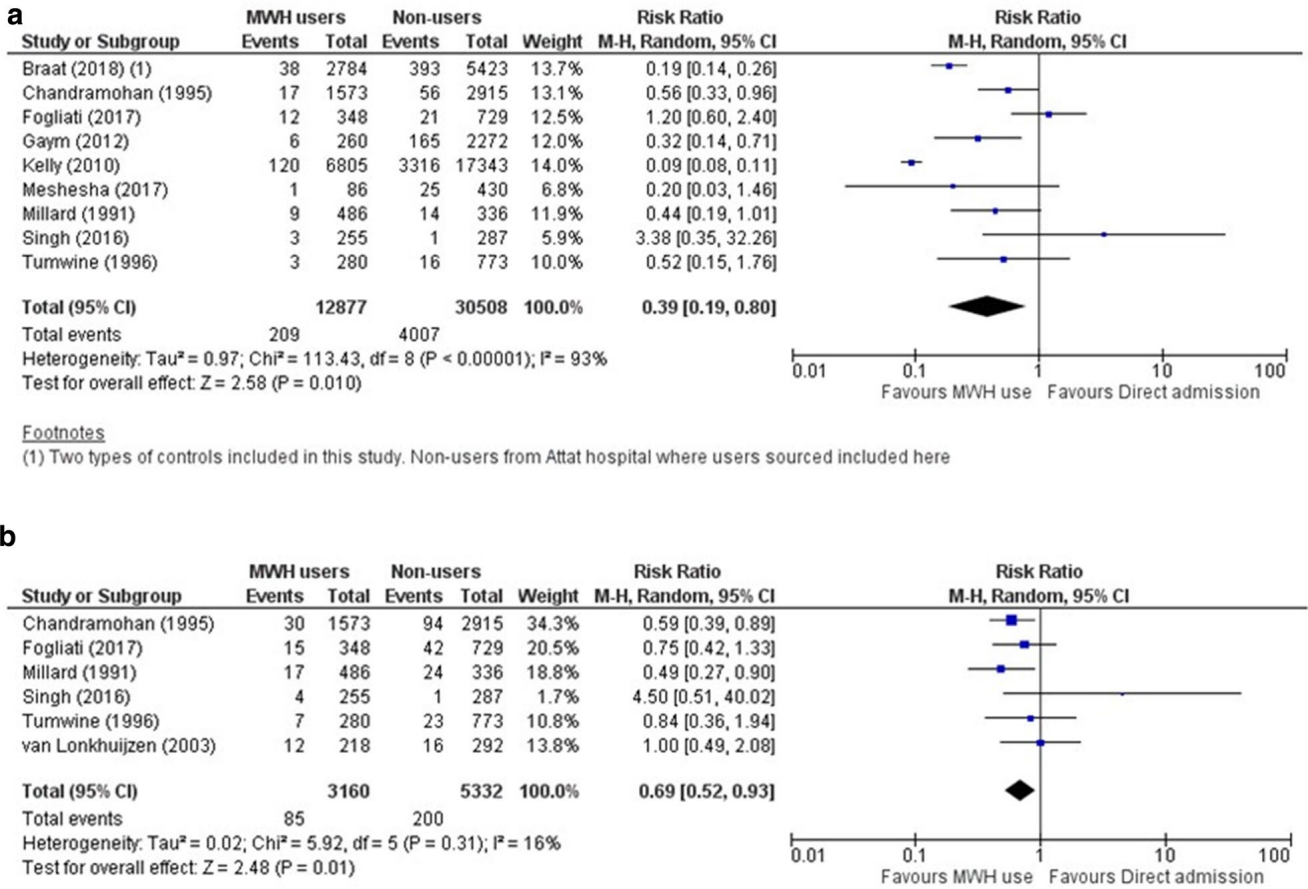
Forest plots of association between MWH use and (a) stillbirths and (b) perinatal mortality

The pooled estimates are suggestive of an association between MWH use and lower stillbirths (pooled Risk Ratio [RR] = 0.39, 95% Confidence Interval [CI]: 0.19 to 0.80; nine studies; 43,385 participants) and to a lesser extent lower perinatal mortality (pooled RR = 0.69, 95% CI: 0.52 to 0.93; six studies; 8,492 participants) (Fig. 1). The comparative similarity in weights calculated for stillbirths point to higher between-studies than within-study variance [29]; this is also reflected in the high values of I^2^ (I^2^ = 93%, indicating 93% of the total variation is attributable to heterogeneity [33]) and τ^2^ (τ^2^ = 0.97).

The lower I^2^ values suggest that there is more consistency among studies conducted in Ethiopia (I^2^ = 86%) and even more among those conducted in other countries (I^2^ = 35%) than when all studies are considered together (I^2^ = 93%) (Table 2).

**Table 2:** Results of sub-group analyses

Sub-groups		Relative Risk (95% confidence interval)	I^2 (^%)	τ^2^	Number of studies	Number of participants	Test for subgroup differences (*p*-value)
Country	Ethiopia	0.17 (0.09–0.31)	86	0.28	4	35,403	< 0.001
	Other country	0.70 (0.43–1.14)	35	0.10	5	7982	
Managing authority	Government	0.62 (0.20–1.90)	42	0.47	3	5004	0.34
	Non-government	0.32 (0.15–0.70)	94	0.83	6	37,839	

Overall, the reduction in the between-study variance for country sub-groups (τ^2^ = 0.10—0.28 subgroups versus τ^2^ = 0.97 all studies) suggests that between-country contextual differences could be one source of heterogeneity. The between-study variance was also lower when the type of managing authority was considered. There was more consistency among studies with government-run facilities (I^2^ = 42% τ^2^ = 0.47) than overall (I^2^ = 93% τ^2^ = 0.97). While the test for subgroup differences was not statistically significant, the existence of heterogeneity due to managing authority cannot be ruled out.

## Conclusion

Given the complexity of MWH interventions and the variation in contextual factors, heterogeneity must be appropriately addressed when conducting meta-analysis on MWH effects. More robustly designed studies with adequate reporting are needed to enable exploration of heterogeneity in effects. Careful consideration of the quality of evidence and specific conditions required to improve outcomes for women and babies is required before implementing further scale-up of MWHs.

## Limitations

Firstly, meta-analyses produce “observational” results even if randomized controlled-trials (RCTs) are included as random allocation is not preserved [43]. Observational studies, where assignment to comparison groups is not random, are considered to be at even higher risk for selection bias and confounding than RCTs [34]. While a random effects model is more suitable for MWH studies, the pooled estimates presented here may still be compromised by bias and confounding inherent to observational designs. Future analyses may consider meta-regression to assess the effect of study-level covariates on effect sizes [28] when at least ten studies are available [30]. If available, adjusted analyses with comparable adjustment variables can also be used to generate adjusted pooled estimates. Ideally, however, additional individual studies using robust designs are required for results from meta-analyses to be more informative. RCTs are generally accepted as providing the highest quality evidence [34] if well designed, conducted and reported. Where it is not feasible or ethical to conduct trials, longitudinal studies with careful participant selection, adequate confounder information, sufficient follow-up levels that analyse data suitably may be acceptable alternatives. Availability of additional studies would also improve estimates of between-study variance (τ^2^) which tend to be imprecise with fewer available studies [28]. Precision, in random effects models, is enhanced by the number of studies included, not study sample sizes [29].

Secondly, while there is an urgent need to improve methodological reporting in primary studies as illustrated in Table 1, there is an equal necessity to provide more details about MWH models themselves. Specifically, information on referral criteria and practices, community outreach activities to raise awareness and facilitate women’s access to MWHs, duration of stay and gestational age at admission, accommodation services available at MWHs, associated costs, level of monitoring of MWHs by health workers, the stage of labour when women are transferred to the health facility, and level of obstetric care available are needed to have a clear understanding of what is required to achieve reported reductions in mortality. This information could support a more comprehensive exploration of heterogeneity which we were not able to do due to the small number of studies and insufficient reporting in individual studies.

Thirdly, a better understanding of modifiable risk factors associated with stillbirths and neonatal deaths is required to assess the extent to which MWHs could potentially facilitate improved perinatal outcomes. A study investigating modifiable health-system risk factors reported that having to wait more than 10 min to receive care after reaching a facility was associated with higher odds of stillbirth [44]. Other modifiable risk factors for stillbirths include maternal infections and prolonged pregnancy [45] which may be addressed through quality antenatal and intrapartum care, irrespective of MWH use. Reporting the type of stillbirth (intrapartum or antepartum) in future studies may help to disentangle stillbirths that can be averted through access to timely obstetric care (intrapartum stillbirths) and those which result from more long-term issues such as foetal growth restriction [45]. Only one of the studies included in the review made this distinction [21] making it impossible to explore.

Stillbirths and neonatal deaths are also a relatively rare event, which would make it difficult for studies with small sample sizes to detect meaningful changes in outcomes. Any reported associations between MWH use and stillbirth rates or perinatal mortality should, thus, be interpreted with caution.

A defining feature of systematic reviews is the use of clearly articulated, well-documented, comprehensive search strategies targeting multiple sources that are designed to capture the highest proportion of eligible studies in a transparent and reproducible fashion. In this way, bias is minimized and more reliable estimates are generated [30]. Since our aim was to illustrate the issues associated with statistical modelling, we did not repeat the search but largely relied on studies identified by Bekele and colleagues [11].

Finally, no firm conclusions can be drawn about the effectiveness of MWHs in reducing perinatal mortality from meta-analyses that do not employ methods that appropriately incorporate contextual variation and adequately consider the quality of included studies. The need to update evidence on MWH effectiveness using well-designed studies from diverse settings that reflect current levels of service use and quality remains.

## Data Availability

Not applicable.

## Abbreviations

CI: Confidence interval
MWH: Maternity waiting homes
RR: Relative risk

## References

[1] World Health Organization (1996). Maternity waiting homes: a review of experiences.

[2] Ministry of Health Ethiopia (2015). Guideline for the establishment of standardized maternity waiting homes at health centres/facilities.

[3] World Health Organization (2015). WHO Recommendations on health promotion interventions for maternal and newborn health.

[4] Wild K, Kelly P, Barclay L, Martins N (2015). Agenda setting and evidence in maternal health: connecting research and policy in timor-leste. Front Public Heal.

[5] Lori JR, Perosky JE, Rominski S, Munro-Kramer ML, Cooper F, Kofa A (2020). Maternity waiting homes in Liberia: Results of a countrywide multi-sector scale-up. PLoS ONE.

[6] Lori JR, Perosky J, Munro-kramer ML, Veliz P, Musonda G, Kaunda J (2019). Maternity waiting homes as part of a comprehensive approach to maternal and newborn care: a cross-sectional survey. BMC Pregnancy Childbirth..

[7] Braat F, Vermeiden T, Getnet G, Schiffer R, van den Akker T, Stekelenburg J (2018). Comparison of pregnancy outcomes between maternity waiting home users and non-users at hospitals with and without a maternity waiting home: retrospective cohort study. Int Health.

[8] Kelly J, Kohls E, Poovan P, Schiffer R, Redito A, Winter H (2010). The role of a maternity waiting area (MWA) in reducing maternal mortality and stillbirths in high-risk women in rural Ethiopia. BJOG.

[9] van Lonkhuijzen L, Stekelenburg J, van Roosmalen J (2012). Maternity waiting facilities for improving maternal and neonatal outcome in low-resource countries. Cochrane database Syst Rev..

[10] Buser JM, Lori JR (2016). Newborn outcomes and maternity waiting homes in low and middle-income countries: a scoping review. Matern Child Health J..

[11] Bekele BB, Dadi TL, Tesfaye T (2019). The significant association between maternity waiting homes utilization and perinatal mortality in Africa: systematic review and meta-analysis. BMC Res Notes..

[12] Bonawitz R, Mcglasson KL, Kaiser JL, Ngoma T, Fong RM, Biemba G (2019). Quality and utilization patterns of maternity waiting homes at referral facilities in rural Zambia : a mixed-methods multiple case analysis of intervention and standard of care sites. PLoS ONE.

[13] Getachew B, Liabsuetrakul T, Gebrehiwot Y (2019). Association of maternity waiting home utilization with women’s perceived geographic barriers and delivery complications in Ethiopia. Int J Heal Plan Manag..

[14] Kaiser JL, Fong RM, Ngoma T, Mcglasson KL, Biemba G, Hamer DH (2019). The effects of maternity waiting homes on the health workforce and maternal health service delivery in rural Zambia:a qualitative analysis. Hum Resour Health..

[15] Perosky JE, Lockhart MLMN, Musonda GK, Naggayi A, Lori JR (2019). Maternity waiting homes as an intervention to increase facility delivery in rural Zambia. Int J Gynaecol Obstet..

[16] Pujihartati SH, Wijaya M, Demartoto A (2020). The importance of socializing maternity waiting home in the attempt of reducing maternal mortality rate in wonogiri regency. Adv Soc Sci Educ Humanit Res.

[17] Tiruneh GT, Getu YN, Abdukie MA, Eba GG, Keyes E, Bailey PE (2019). Distribution of maternity waiting homes and their correlation with perinatal mortality and direct obstetric complication rates in Ethiopia. BMC Pregnancy Childbirth..

[18] Coley KM, Perosky JE, Nyanplu A, Kofa A, Anankware JP, Moyer CA (2020). Acceptability and feasibility of insect consumption among pregnant women in Liberia. Matern Child Nutr..

[19] Idris IO, Araoye D, Chijioke OD, Gavkalova N (2020). A Policy Discussion on maternity waiting home in Zambia to achieve its vision 2030 on maternal and perinatal mortality. J Fam Med Heal Care.

[20] Chandramohan D, Cutts F, Millard P (1995). The effect of stay in a maternity waiting homes on perinatal mortality. J Trop Med Hyg.

[21] Fogliati P, Straneo M, Mangi S, Azzimonti G, Kisika F, Putoto G (2017). A new use for an old tool: maternity waiting homes to improve equity in rural childbirth care. Results from a cross-sectional hospital and community survey in Tanzania. Health Policy Plan..

[22] Lori JR, Munro ML, Rominski S, Williams G, Dahn BT, Boyd CJ (2013). Maternity waiting homes and traditional midwives in rural Liberia. Int J Gynecol Obstet.

[23] Meshesha B, Dejene G, Hailemariam T (2017). The role of maternity waiting area in improving obstetric outcomes: a comparative cross-sectional study, Jinka Zonal Hospital, Southern regional state. J Womens Heal Care..

[24] Poovan P, Kifle F, Kwast BE (1990). A maternity waiting home reduces obstetric catastrophes. World Health Forum.

[25] Singh K, Speizer I, Kim ET, Lemani C, Phoya A (2017). Reaching vulnerable women through maternity waiting homes in Malawi. Int J Gynaecol Obstet.

[26] Spaans W, van Roosmalen J, van Wiechen CMA (1998). A maternity waiting home experience in Zimbabwe. Int J Gynecol Obstet.

[27] van Lonkhuijzen L, Stegeman M, Nyirongo R, van Roosmalen J (2003). Use of maternity waiting home in rural Zambia. Afr J Reprod Health.

[28] Borenstein M, Hedges LV, Higgins JPT, Rothstein HR (2009). Introduction to meta-analysis.

[29] Borenstein M, Hedges LV, Higgins JPT, Rothstein HR (2010). A basic introduction to fixed-effect and random-effects models for meta-analysis. Res Synth Method..

[30] Higgins JPT, Chandler J, Cumpston M, Li T, Page M, Welch V (2019). Cochrane handbook for systematic reviews of interventions (version 6.0).

[31] Pigott T (2012). Advances in Meta-Analysis.

[32] Heterogeneity BM (2019). Common mistakes in meta-analysis and how to avoid them.

[33] Higgins JPT (2008). Commentary: Heterogeneity in meta-analysis should be expected and appropriately quantified. Int J Epidemiol.

[34] Metelli S, Chaimani A (2020). Challenges in meta-analyses with observational studies. Evid Based Ment Heal.

[35] Tanner-smith EE, Grant S (2018). Meta-analysis of complex interventions. Annu Rev Psychol.

[36] Penn-Kekana L, Pereira S, Hussein J, Bontogon H, Chersich M, Munjanja S (2017). Understanding the implementation of maternity waiting homes in low- and middle-income countries: a qualitative thematic synthesis. BMC Pregnancy Childbirth..

[37] Higgins JPT, Thompson SG, Spiegelhalter DJ (2009). A re-evaluation of random-effects meta-analysis. J R Stat Soc..

[38] Higgins JPT, López-lópez JA, Becker BJ, Davies SR, Dawson S, Grimshaw JM (2019). Synthesising quantitative evidence in systematic reviews of complex health interventions. BMJ Glob Heal..

[39] Mueller M, Addario MD, Egger M, Cevallos M, Dekkers O, Mugglin C (2018). Methods to systematically review and meta-analyse observational studies: a systematic scoping review of recommendations. BMC Med Res Methodol..

[40] Gaym A, Pearson L, Soe KWW (2012). Maternity waiting homes in Ethiopia-three decades experience. Ethiop Med J.

[41] Millard P, Bailey J, Hanson J (1991). Antenatal village stay and pregnancy outcome in rural Zimbabwe. Cent Afr J Med..

[42] Tumwine JK, Dungare PS (1996). Maternity waiting shelters and pregnancy outcome: experience from a rural area in Zimbabwe. Ann Trop Paediatr.

[43] Viswanathan M, Mcpheeters ML, Murad MH, Butler ME, Beth EE, Dyson MP (2017). AHRQ series on complex intervention systematic reviews paper 4: selecting analytic approaches. J Clin Epidemiol.

[44] Neogi SB, Sharma J, Negandhi P, Chauhan M, Reddy S, Sethy G (2018). Risk factors for stillbirths: how much can a responsive health system prevent ?. BMC Pregnancy Childbirth..

[45] Lawn JE, Blencowe H, Waiswa P, Amouzou A, Mathers C, Hogan D (2016). Stillbirths: rates, risk factors, and acceleration towards 2030. Lancet.

[46] Downes MJ, Brennan ML, Williams HC, Dean RS (2016). Development of a critical appraisal tool to assess the quality of cross-sectional studies (AXIS). BMJ Open..

